# Supplementary material for: Testing the Interface Hypothesis: Evidence from processing directions of possession transfer in double object constructions by L1-Mandarin Chinese L2-English learners
Source: PLoS One. 2025 Feb 12;20(2):e0313965. doi: 10.1371/journal.pone.0313965 (PMC11819539; doi:10.1371/journal.pone.0313965)
Supplement: Attachment — Submitted filename: Response to Reviewers.docx [file pone.0313965.s002.docx]

**Response to Reviewers**

Dear reviewers,

Thank you for your valuable comments and suggestions, which have helped greatly in the improvement of the paper. We have carefully considered each of your comments and have revised the manuscript (highlighted in red), and we also detail below our responses to major points (in italic) raised in the reviews.

*The study mentions L1 transfer as a possible variable influencing L2 learners’ difficulties with DOCs, but this aspect could be explored more thoroughly. Providing a more detailed analysis of how L1 Mandarin structures might interfere with or facilitate the acquisition of English DOCs would enhance the explanatory power of the study.*

We acknowledge your valuable point regarding the influence of L1 transfer on the acquisition of English Double Object Constructions (DOCs) by Chinese L2 learners. In response, we have refined the relevant paragraphs in the manuscript, which can be found on pages 41-42 (lines 759-782). Specifically, we elaborated on how Chinese and English DOCs differ in the direction of possession transfer, particularly distinguishing between rightward and leftward verbs. For example, whereas possession transfer is exclusively rightward in English DOCs, Chinese DOCs can feature both rightward and leftward transfer. Our findings suggest that L2 learners can acquire English DOCs containing rightward verbs relatively early, but they struggle with those containing leftward verbs throughout different proficiency levels, likely due to L1 interference.

By detailing how L1 processing mechanisms either facilitate or interfere with the acquisition of English DOCs depending on the alignment of possession transfer directions, we hope we can address the reviewer’s concern regarding the influence of L1 on L2 acquisition of DOCs.

Best regards,

Tao Zeng
